# Mitochondrial-inflammatory coupling in HFpEF: an emerging mechanistic framework for understanding the cardioprotective effects of SGLT2 inhibitors

**DOI:** 10.3389/fcvm.2026.1881631

**Published:** 2026-07-29

**Authors:** Muhammad Ghani Youniszad, Yongzhi Deng

**Affiliations:** Department of Cardiovascular Surgery, The Affiliated Hospital of Shanxi Medical University, Shanxi Cardiovascular Hospital (Institute), Shanxi Clinical Medical Research Center for Cardiovascular Disease, Taiyuan, Shanxi, China

**Keywords:** cardiac metabolism, HFpEF, immunometabolism, mitochondrial dysfunction, NLRP3 inflammasome, precision medicine, SGLT2 inhibitor

## Abstract

Heart failure with preserved ejection fraction (HFpEF) remains a major cause of cardiovascular morbidity and mortality, yet lacks effective disease-modifying therapies owing to pronounced biological heterogeneity. Emerging evidence indicates that HFpEF is not merely a disorder of diastolic dysfunction but a systemic immunometabolic condition characterized by mitochondrial energetic impairment, altered substrate utilization, and chronic low-grade inflammation. This review examines the potential role of sodium-glucose cotransporter 2 (SGLT2) inhibitors in modulating mitochondrial-inflammatory coupling in HFpEF and proposes this axis as an emerging mechanistic framework that may contribute to their therapeutic effects. We review preclinical and clinical evidence suggesting that HFpEF is associated with coordinated immunometabolic reprogramming across cardiomyocytes, endothelial cells, and cardiac immune populations, which may contribute to mitochondrial-inflammatory uncoupling. We discuss evidence that SGLT2 inhibitors act beyond glycosuria to potentially improve myocardial energetic efficiency, enhance mitochondrial quality control, reduce oxidative stress, and attenuate maladaptive inflammatory signaling, including inflammasome activation. Collectively, these findings suggest that restoration of mitochondrial-inflammatory coupling may partly explain the clinical benefits of SGLT2 inhibitors in HFpEF, although direct mechanistic evidence in humans remains limited. Finally, we highlight emerging biomarkers and future therapeutic strategies targeting the mitochondrial-inflammator*y* axis to support the development of precision medicine approaches for HFpEF.

## 1 Introduction

Heart failure with preserved ejection fraction (HFpEF) accounts for approximately half of all heart failure cases and represents a growing cause of morbidity and mortality. Its prevalence is increasing, particularly among older adults (1, 2). Despite the increasing prevalence and substantial morbidity and mortality, HFpEF has remained challenging to treat with effective disease-modifying therapies, reflecting its marked biological heterogeneity and the incomplete understanding of its underlying pathophysiological mechanisms (3–5). Importantly, exercise intolerance, a cardinal clinical manifestation of HFpEF, is increasingly recognized as the consequence of integrated abnormalities involving the heart, vasculature, skeletal muscle, autonomic regulation, and peripheral oxygen utilization rather than isolated myocardial dysfunction alone. This systems-level perspective further supports the concept that biological heterogeneity is a defining feature of HFpEF and underscores the need for phenotype-oriented mechanistic frameworks (6, 7).

The accumulation of experimental and clinical evidence has increasingly supported the view that HFpEF may represent an immunometabolic syndrome characterized by mitochondrial dysfunction and chronic low-grade inflammation (8, 9). Obesity, type 2 diabetes, aging, and chronic kidney disease are common comorbidities among HFpEF patients and are associated with impaired mitochondrial energy production, altered substrate utilization, and increased production of reactive oxygen species. The metabolic disturbances may contribute to inflammatory activation in cardiomyocytes, endothelial cells, and cardiac immune populations, potentially promoting microvascular dysfunction, myocardial stiffness, and exercise intolerance (10). Although mitochondrial dysfunction and chronic inflammation appear to be closely interconnected in HFpEF, their precise causal relationship and relative contributions to disease progression remain under active investigation (11, 12).

Sodium-glucose cotransporter-2 inhibitors (SGLT2 inhibitors) have demonstrated significant reductions in heart failure hospitalization and improvements in clinical outcomes in patients with HFpEF in several large-scale clinical trials, regardless of diabetes status (13, 14). These findings represent a major therapeutic advance for a condition in which previous treatment strategies have shown limited success. Although the mechanisms underlying these clinical benefits remain incompletely understood, they are unlikely to be explained solely by glucose lowering, natriuresis, or hemodynamic effects (15, 16). Consequently, increasing attention has focused on potential mechanisms involving cardiac metabolism, mitochondrial function, and inflammatory signaling pathways (17, 18).

Recent studies suggest that SGLT2 inhibitors may influence myocardial energy metabolism and may exert indirect anti-inflammatory effects, leading to the hypothesis that they can modulate mitochondrial-inflammatory interactions (19, 20). Preclinical studies have shown that SGLT2 inhibitors may improve substrate utilization, enhance mitochondrial bioenergetic efficiency, reduce reactive oxygen species generation, and attenuate oxidative stress. In parallel, experimental evidence suggests that these agents may attenuate inflammasome activation and pro-inflammatory cytokine signaling in several cardiac and vascular cell types (21, 22). However, much of the mechanistic evidence supporting these effects is derived from experimental models, whereas direct mechanistic validation in humans remains limited. Collectively, these findings have led to the hypothesis that SGLT2 inhibitors may help restore aspects of mitochondrial and inflammatory homeostasis that are disrupted in HFpEF (22, 23).

Unlike previous reviews that have discussed mitochondrial dysfunction, inflammation, or SGLT2 inhibitors separately, this review integrates these interconnected processes within a unified mechanistic framework while critically evaluating the current evidence, its limitations, and remaining knowledge gaps. We propose that the interplay between mitochondrial dysfunction and inflammation may represent an important pathophysiological axis in HFpEF and explore whether modulation of this axis may contribute to the therapeutic effects of SGLT2 inhibitors. We synthesize available preclinical, translational, and clinical evidence, discuss similarities and differences in immunometabolic remodeling between HFpEF and heart failure with reduced ejection fraction (HFrEF), while acknowledging that these syndromes share several overlapping biological mechanisms, and examine emerging biomarkers and phenotyping strategies that may facilitate more individualized therapeutic approaches. Rather than presenting mitochondrial-inflammatory coupling as an established mechanism, this review aims to provide an evidence-based conceptual framework to guide future mechanistic investigations and precision medicine strategies in HFpEF.

## 2 HFpEF as a systemic immunometabolic-mitochondrial disorder

**Figure 1:**
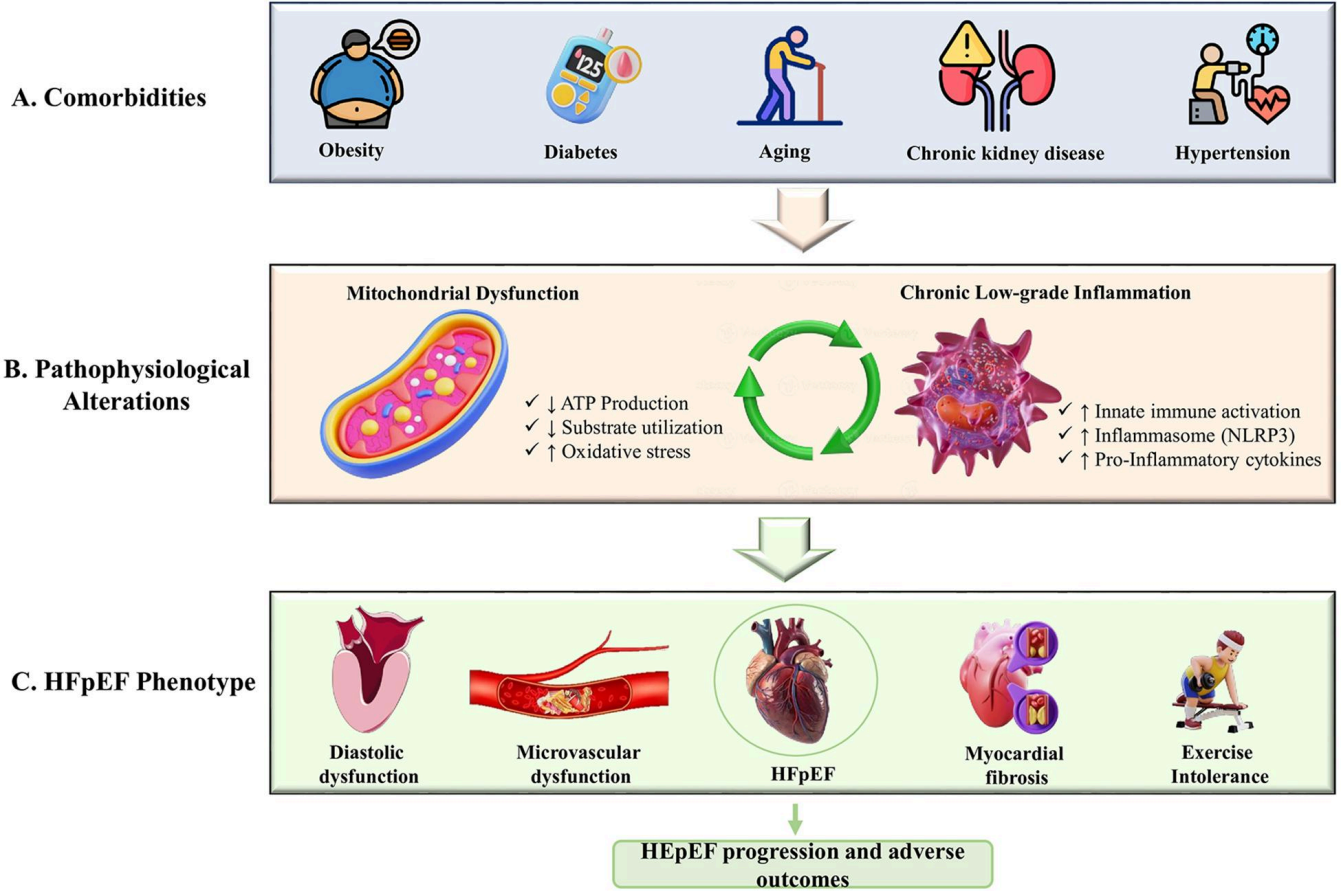
Multilayered immunometabolic framework underlying HFpEF pathogenesis. The condition of heart failure with preserved ejection fraction (HFpEF) results from the combination of multiple systemic cardiometabolic conditions, which include obesity, diabetes, aging, and chronic kidney disease. The cardiovascular tissues experience a continuous metabolic battle because of these conditions. The central disease pathway develops through two main processes, which include mitochondrial dysfunction and ongoing mild body inflammation. The body uses its metabolic pathways to create energy while producing harmful mitochondrial byproducts, which enable the immune system to respond and activate inflammatory pathways. The two systems work together to create a unique HFpEF condition, which shows diastolic heart failure, microvascular damage, heart muscle rigidity, and decreased ability to exercise. The hierarchical structure demonstrates that HFpEF functions as an entire body immunometabolic condition, which results from the combined effects of mitochondrial dysfunction and inflammatory system disturbances, instead of just heart functions that are not working properly.

HFpEF is increasingly recognized as a systemic syndrome extending beyond isolated cardiac dysfunction. Unlike heart failure with reduced ejection fraction (HFrEF), which is often characterized by more extensive cardiomyocyte loss and structural remodeling, HFpEF appears to be more strongly influenced by metabolic stress, immune system dysregulation, and systemic mitochondrial dysfunction, although these mechanisms contribute to varying degrees across both syndromes (8, 24, 25). This systemic pathophysiology contributes to the clinical heterogeneity of HFpEF and may partly explain its limited responsiveness to conventional heart failure therapies (as shown in Figure 1).

### 2.1 From diastolic dysfunction to immunometabolic disease

The traditional view of HFpEF as an isolated disorder of diastolic function does not fully explain its heterogeneous clinical manifestations, and has not been able to account for the various manifestations of the disease, as demonstrated by the studies conducted on the patients (26–28). Obesity, diabetes, age, hypertension, chronic kidney disease, and systemic inflammation are common comorbidities associated with HFpEF and are thought to contribute importantly to disease development, particularly in cardiometabolic HFpEF phenotypes. Which is engaged in immune system metabolism and, as a result, disrupts cell energy and maintains low-grade inflammation at the level of communication (29, 30).

HFpEF is increasingly viewed as an immunometabolic syndrome in many patients, although considerable biological heterogeneity exists. While inflammatory signals damage the integrity and energy-producing efficiency of the mitochondria, metabolic stress alters the immune cells' makeup and activity (30, 31). In the case of HFpEF, this reciprocal interaction may establish a self-reinforcing cycle that contributes to myocardial stiffness, microvascular dysfunction, and exercise intolerance. Importantly, HFpEF represents a heterogeneous clinical syndrome encompassing multiple biological phenotypes rather than a single disease entity. In addition to cardiometabolic HFpEF associated with obesity, diabetes, aging, and chronic inflammation, patients meeting HFpEF diagnostic criteria may include those with infiltrative cardiomyopathies, significant valvular heart disease, pulmonary vascular disease, or other HFpEF mimics. Consequently, the mitochondrial-inflammatory framework proposed in this review is likely to be most applicable to cardiometabolic HFpEF, where metabolic dysfunction and chronic low-grade inflammation are considered major drivers of disease progression. Consistent with emerging conceptual models, HFpEF should be viewed as a heterogeneous clinical syndrome in which exercise limitation arises from integrated cardiac and extracardiac mechanisms. Besides impaired diastolic reserve, endothelial dysfunction, pulmonary vascular abnormalities, skeletal muscle mitochondrial dysfunction, reduced peripheral oxygen extraction, autonomic dysregulation, and systemic metabolic disturbances collectively contribute to impaired exercise performance. Consequently, the relative contribution of mitochondrial-inflammatory interactions is expected to differ among individual HFpEF phenotypes.

### 2.2 Mitochondrial dysfunction as an important pathophysiological component

As the primary source of cellular energy and an important regulator of redox and inflammatory signaling, mitochondria are increasingly recognized as contributors to HFpEF pathophysiology. In HFpEF, cardiac mitochondria may exhibit increased reactive oxygen species (ROS) production, impaired metabolic flexibility, and reduced oxidative phosphorylation (32). Similar alterations have also been reported in vascular endothelial cells, fibroblasts, and immune cells, supporting the view that HFpEF involves coordinated metabolic disturbances across multiple cardiovascular and extracardiac cell types (33).

Comorbidities such as obesity, type 2 diabetes, aging, and chronic kidney disease may disrupt myocardial substrate utilization, reduce metabolic flexibility, and promote accumulation of incompletely oxidized metabolic intermediates. These changes may impair adenosine triphosphate (ATP) generation and increase cellular energetic stress (34). Defects and mitochondrial biogenesis may further promote the persistence of damaged mitochondria. Consequently, mitochondrial dysfunction may impair cardiac energy metabolism while simultaneously enhancing redox-sensitive and inflammatory signaling through several interconnected mechanisms (35).

### 2.3 Inflammation as a metabolic consequence and driver

In HFpEF, inflammation may arise both as a consequence of mitochondrial dysfunction and as a contributor to further metabolic impairment (36). Excessive mitochondrial ROS production, release of mitochondrial DNA, and altered redox signaling may activate innate immune pathways, including the NOD-like receptor pyrin domain-containing 3 (NLRP3) inflammasome (37, 38). These processes may promote the production of inflammatory cytokines such as interleukin-1β and interleukin-6, which can exert local and systemic effects on myocardial structure, vascular function, and cellular metabolism (38).

Inflammation in cardiometabolic HFpEF is generally characterized as chronic, low-grade, and metabolically driven rather than acute or infection-related. This distinction may partly explain why broadly targeted anti-inflammatory therapies have shown variable or limited benefits in unselected HFpEF populations (39, 40). Inflammatory signaling may therefore represent both a downstream response to mitochondrial and metabolic stress and an active contributor to persistent energetic dysfunction, endothelial injury, extracellular matrix remodeling, and impaired myocardial relaxation (40).

### 2.4 Immunometabolic reprogramming

A proposed feature of HFpEF is coordinated immunometabolic reprogramming across multiple cardiac and vascular cell types. However, the extent and biological relevance of this remodeling are likely to vary among HFpEF phenotypes and may be less prominent in non-cardiometabolic forms of the syndrome. In cardiomyocytes, impaired mitochondrial ATP production and increased oxidative stress may contribute to abnormal calcium handling and incomplete myocardial relaxation (41). In endothelial cells, mitochondrial dysfunction may reduce nitric oxide bioavailability, impair coronary microvascular reserve, and promote endothelial inflammatory activation. Cardiac macrophages and other immune-cell populations may also shift toward more pro-inflammatory phenotypes and contribute to cytokine production, fibrosis, and extracellular matrix remodeling (42).

This pattern of immunometabolic remodeling may differ quantitatively between HFpEF and heart failure with reduced ejection fraction (HFrEF), although mitochondrial dysfunction, inflammatory activation, and metabolic abnormalities contribute to both syndromes. In many cardiometabolic HFpEF phenotypes, functional impairment occurs despite relatively preserved myocardial architecture, suggesting that systemic metabolic stress, microvascular dysfunction, and inflammatory signaling may have particularly important roles. These differences may have therapeutic implications and support further evaluation of metabolism- and inflammation-directed strategies in biologically defined HFpEF subgroups (43).

### 2.5 Mitochondrial-inflammatory uncoupling in HFpEF

Under physiological conditions, mitochondrial energetic status and inflammatory signaling are closely coordinated, allowing cells to adjust immune responses according to substrate availability, redox state, and energetic demand. In HFpEF, this coordination may become dysregulated. Persistent inflammatory signaling can impair mitochondrial function, whereas defective mitochondrial energetics and excessive ROS production may further sustain innate immune activation (44). His bidirectional interaction has been proposed as a potentially important pathogenic feature of cardiometabolic HFpEF.

Within this framework, mitochondrial–inflammatory uncoupling refers to a maladaptive state in which impaired mitochondrial energetics coexist with persistent inflammatory activation and defective resolution. This concept may help explain why interventions directed toward a single downstream pathway, such as fibrosis, hemodynamic burden, or inflammation alone, have often produced incomplete benefits in heterogeneous HFpEF populations. Therapeutic strategies that simultaneously improve mitochondrial function and attenuate maladaptive inflammatory signaling may therefore offer advantages over isolated pathway-specific approaches, although this hypothesis requires prospective clinical validation (45).

Viewing HFpEF as a multisystem immunometabolic disorder also shifts attention beyond cardiac mechanics alone. This perspective highlights the potential importance of restoring metabolic flexibility, mitochondrial quality control, redox homeostasis, and coordinated immune-metabolic regulation (46). It may also provide one possible mechanistic explanation for the clinical effects of sodium–glucose cotransporter 2 (SGLT2) inhibitors, which have been proposed to influence pathways related to energy metabolism, mitochondrial function, oxidative stress, and inflammatory signaling. However, the relative contribution of each pathway remains incompletely understood.

SGLT2 inhibitors may partly exert their therapeutic effects by modulating upstream processes associated with mitochondrial–inflammatory interactions. This integrated, systems-level mode of action may contribute to the consistent clinical benefits observed across several HFpEF trials, although direct mechanistic confirmation in human HFpEF remains limited (47).

## 3 Mitochondrial-inflammatory uncoupling: an emerging mechanistic framework in HFpEF

Inflammatory activation is closely linked to cellular metabolic state, as immune responses are physiologically regulated according to energy availability and mitochondrial function. Mitochondria are increasingly recognized as important regulators of inflammatory and energetic responses through redox signaling, substrate availability, and innate immune activation (10, 48). Emerging evidence suggests that disruption of this regulatory balance may contribute to a state of mitochondrial-inflammatory uncoupling in HFpEF, characterized by persistent inflammatory signaling alongside impaired mitochondrial energetics.

In the healthy heart, mitochondrial oxidative phosphorylation supports ATP-dependent cellular functions while minimizing the production of reactive oxygen species (ROS). Through redox-sensitive signaling pathways, mitochondria help coordinate inflammatory activation while maintaining cellular homeostasis (49). Obesity, insulin resistance, and aging may promote metabolic stress in HFpEF, leading to impaired substrate utilization, mitochondrial metabolic dysfunction, and increased ROS production. These metabolic alterations may sustain activation of innate immune pathways, including inflammasome signaling, thereby limiting adaptive inflammatory resolution (50).

One proposed feature of mitochondrial–inflammatory uncoupling in cardiometabolic HFpEF is impaired resolution of chronic inflammatory signaling. Cytosolic pattern-recognition receptors may be activated by mitochondrial-derived danger-associated molecular patterns, including oxidized mitochondrial DNA (mtDNA) and cardiolipin, thereby contributing to persistent inflammasome activation even in the absence of acute tissue injury. Persistent inflammatory signaling may also impair mitophagy and mitochondrial biogenesis, potentially contributing to progressive mitochondrial dysfunction (19). This proposed framework integrates several previously independent observations in HFpEF, including mild structural remodeling, exercise intolerance, microvascular dysfunction, and systemic inflammation. Collectively, these observations suggest that mitochondrial–inflammatory interactions may partly contribute to the development and progression of HFpEF, particularly in patients with cardiometabolic phenotypes.

Within this conceptual framework, impaired myocardial energetics, together with inflammation-mediated alterations in myocardial relaxation, calcium handling, and vascular function, may contribute to exercise intolerance despite preserved systolic function. However, contemporary evidence suggests that exercise intolerance in HFpEF reflects integrated dysfunction across multiple organ systems rather than impaired myocardial energetics alone. Alterations in endothelial function, skeletal muscle metabolism, peripheral oxygen extraction, pulmonary vascular reserve, and systemic metabolic regulation collectively interact with cardiac abnormalities to determine functional capacity. Within this broader framework, mitochondrial–inflammatory dysregulation may be interpreted as one potential contributor to the multisystem pathophysiology underlying exercise intolerance rather than its sole determinant (51).

Although increasing experimental evidence supports interactions between mitochondrial dysfunction and chronic inflammation in HFpEF, direct clinical validation of mitochondrial–inflammatory uncoupling as a dominant pathogenic mechanism remains limited. Most mechanistic insights are derived from experimental models, whereas human studies have largely demonstrated associations rather than causality. Consequently, the mitochondrial-inflammatory framework should be viewed as an evolving conceptual model that integrates current evidence while highlighting important areas for future mechanistic and translational investigation.

## 4 Immunometabolic reprogramming across cardiac and vascular cell types

In HFpEF, proposed mitochondrial-inflammatory uncoupling is not limited to cardiomyocytes but may involve coordinated immunometabolic remodeling across multiple cardiac and vascular cell types (Table 1) (52, 53).

**Table 1:** Immunometabolic and mitochondrial alterations across cell types in HFpEF.

Cell type	Metabolic alterations	Mitochondrial defects	Inflammatory consequences	Functional impact
Cardiomyocytes	↓ FAO, ↓ Ketone use, metabolic inflexibility	↓ ATP Efficiency, ↑ ROS, Impaired Ca^2+^ handling	NF-kB activation, inflammasome priming	Diastolic dysfunction
Endothelial cells	Altered glucose & FA metabolism	↓ NO signaling, mtROS	Endothelial inflammation	Microvascular dysfunction
Cardiac macrophages	Shift to glycolytic/pro-inflammatory metabolism	Mitochondrial rewiring	Cytokine release (IL-6, TNF-α)	Fibrosis, ECM remodeling
Fibroblasts	Metabolic activation	Altered mitochondrial signaling	Pro-fibrotic cytokines	Stiff myocardium

### 4.1 Cardiomyocytes

Metabolic inflexibility of cardiomyocytes is increasingly recognized as an important feature of HFpEF. Under conditions of metabolic stress, cardiomyocytes may exhibit altered substrate utilization characterized by reduced metabolic flexibility and impaired fatty acid oxidation, with variable compensatory increases in glucose utilization depending on disease stage and metabolic context. These metabolic alterations may reduce ATP generation and impair myocardial energetics (23, 54). Impaired mitochondrial function may contribute to abnormal calcium handling and incomplete myocardial relaxation. Increased mitochondrial ROS may activate redox-sensitive transcriptional pathways that contribute to inflammatory signaling. Compared with HFrEF, HFpEF generally exhibits less cardiomyocyte loss and structural remodeling, although mitochondrial dysfunction, inflammation, and metabolic abnormalities contribute to both syndromes (55).

### 4.2 Endothelial cells and microvasculature

Endothelial mitochondrial dysfunction is increasingly recognized as an important contributor to HFpEF pathophysiology. Impaired mitochondrial signaling may contribute to reduced nitric oxide bioavailability, increased endothelial inflammation, and disruption of coronary microvascular function (56). These alterations may increase myocardial energetic stress while reducing coronary microvascular perfusion reserve. Furthermore, endothelial inflammation may promote leukocyte recruitment, cytokine production, and amplification of local inflammatory responses, thereby further contributing to microvascular dysfunction (57).

### 4.3 Cardiac immune cells

Experimental evidence suggests that cardiac macrophages and other resident immune cells undergo metabolic reprogramming in HFpEF, promoting more pro-inflammatory phenotypes associated with altered mitochondrial metabolism (58). In HFpEF, immune activation appears to be driven predominantly by metabolic disturbances rather than infectious stimuli. These cells may release cytokines that contribute to fibrosis, extracellular matrix remodeling, and vascular dysfunction. Collectively, these observations support a potential role for immunometabolic integration in sustaining chronic low-grade inflammation in HFpEF, although further mechanistic validation in humans is required (59).

It should be noted that most evidence describing cell-specific immunometabolic remodeling has been derived from experimental and *ex vivo* studies, whereas direct validation in human HFpEF tissues remains comparatively limited. Furthermore, the magnitude and biological relevance of these alterations may differ across distinct HFpEF phenotypes. Future studies integrating single-cell transcriptomics, spatial biology, and detailed clinical phenotyping will be important to clarify the contribution of individual cell populations to disease progression, patient heterogeneity, and therapeutic response.

## 5 SGLT2 inhibitors as modulators of mitochondrial energetics beyond glycosuria

The glucose-lowering and natriuretic effects of sodium–glucose cotransporter 2 (SGLT2) inhibitors alone may not fully explain their clinical benefits in HFpEF. Accumulating experimental and translational evidence suggests that SGLT2 inhibitors may influence mitochondrial metabolism in the heart and other tissues through multiple complementary mechanisms (60). SGLT2 inhibition has been proposed to improve metabolic efficiency, in part by promoting adaptive changes in systemic substrate utilization, including increased ketone body availability and utilization. Ketone bodies may serve as an energetically efficient substrate for the myocardium under selected physiological and pathological conditions, potentially improving myocardial energetics while reducing oxidative stress, although the relative contribution of this mechanism to the clinical benefits of SGLT2 inhibitors remains under investigation (61). These metabolic adaptations may improve myocardial energetics and reduce mitochondrial ROS production, potentially contributing to improved cardiac function in HFpEF.

Preclinical studies suggest that SGLT2 inhibitors may enhance mitophagy and mitochondrial biogenesis, two key components of mitochondrial quality control. Improved mitochondrial quality control may facilitate restoration of redox homeostasis and reduce the release of pro-inflammatory mitochondrial signals. Similar findings have been reported in experimental studies involving cardiomyocytes, endothelial cells, and immune cells, although direct confirmation in patients with HFpEF remains limited. Collectively, these findings suggest that similar mitochondrial regulatory mechanisms may operate across multiple cardiovascular cell types. Available evidence further suggests that SGLT2 inhibitors may attenuate metabolically driven inflammatory signaling by improving mitochondrial function, although the relative importance of direct myocardial vs. systemic metabolic effects remains incompletely understood. These observations support the hypothesis that modulation of mitochondrial function may represent one potential mechanism contributing to the clinical benefits of SGLT2 inhibitors in HFpEF (61–63).

Despite encouraging mechanistic evidence, several uncertainties remain. Most studies investigating the effects of SGLT2 inhibitors on mitochondrial quality control, substrate utilization, and inflammatory signaling have been conducted in experimental models, whereas direct mechanistic evidence in patients with HFpEF remains comparatively limited. Furthermore, the relative contributions of myocardial and systemic metabolic effects have not yet been fully defined. Future mechanistic clinical studies integrating advanced metabolic imaging, biomarker profiling, tissue-based analyses, and phenotype-specific patient stratification will be important to clarify the relative contribution of these pathways to the therapeutic effects of SGLT2 inhibitors in HFpEF.

## 6 Anti-inflammatory actions of SGLT2 inhibitors: evidence and boundaries

Although the magnitude of their anti-inflammatory effects varies across studies, several clinical investigations have reported modest reductions in circulating inflammatory biomarkers following treatment with SGLT2 inhibitors (64, 65). Preclinical studies have consistently demonstrated attenuation of inflammasome activation, reduced cytokine production, and decreased oxidative stress-associated inflammatory signaling following SGLT2 inhibitor treatment. However, direct mechanistic confirmation of these effects in patients with HFpEF remains comparatively limited. Current evidence suggests that the observed anti-inflammatory effects are more likely to be secondary to improvements in metabolic homeostasis and mitochondrial function than to direct immunosuppressive actions, although the relative contribution of these mechanisms remains incompletely understood (65).

This distinction has important therapeutic implications. Rather than acting as conventional immunosuppressive agents, SGLT2 inhibitors may help restore immune–metabolic homeostasis by improving underlying metabolic disturbances that contribute to chronic low-grade inflammation. Such indirect effects may be particularly relevant in cardiometabolic HFpEF phenotypes characterized by metabolic dysfunction and persistent low-grade inflammation. However, direct comparisons with established anti-inflammatory therapies remain limited and require further investigation (66).

Several important questions remain unresolved, including the influence of HFpEF phenotypic heterogeneity, the temporal sequence of immunometabolic adaptations, and the relative contribution of cell type-specific mechanisms. These knowledge gaps highlight the need for integrated mechanistic studies combining inflammatory profiling, mitochondrial functional assessment, advanced metabolic phenotyping, and clinical characterization to better define how SGLT2 inhibitors influence mitochondrial-inflammatory interactions in HFpEF (67).

It should also be acknowledged that reductions in circulating inflammatory biomarkers have not been consistently observed across clinical studies, and the relationship between biomarker changes and improvements in clinical outcomes remains incompletely understood. Furthermore, whether the reported anti-inflammatory effects primarily reflect direct myocardial actions, vascular effects, or indirect systemic metabolic adaptations remains uncertain. Addressing these questions will require prospective mechanistic clinical studies capable of establishing stronger links between immunometabolic modulation and therapeutic benefit while accounting for the biological heterogeneity of HFpEF.

## 7 Restoration of mitochondrial-inflammatory coupling: an emerging mechanistic framework

**Figure 2:**
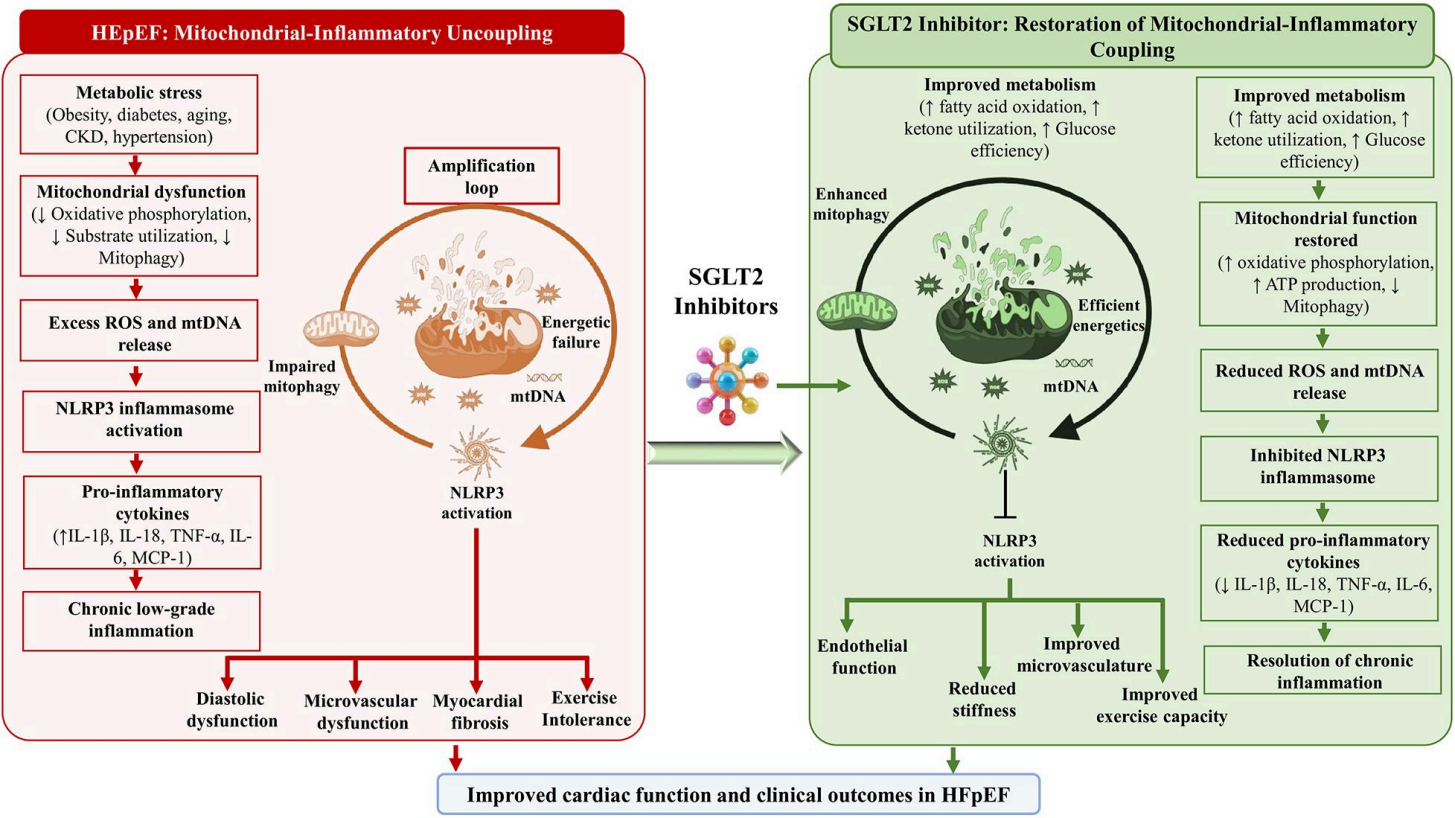
Proposed mechanistic framework for mitochondrial-inflammatory interactions in HFpEF and potential modulation by SGLT2 inhibitors. Schematic illustration of the proposed interactions between metabolic stress, mitochondrial dysfunction, and chronic inflammatory signaling in HFpEF. The left panel demonstrates how cardiometabolic risk factors, including obesity, diabetes, aging, and chronic kidney disease, may contribute to impaired mitochondrial energetics, altered substrate utilization, disrupted mitochondrial quality control, increased reactive oxygen species (ROS) production, and activation of inflammatory pathways, including the NLRP3 inflammasome. These interconnected processes are proposed to contribute to sustained immunometabolic dysregulation in HFpEF. The right panel illustrates the proposed mechanisms through which SGLT2 inhibitors may improve mitochondrial function, enhance substrate utilization and mitochondrial quality control, reduce oxidative stress, and attenuate inflamamtory signaling. These mechanisms may contribute to improvd myocardial energetic efficiency and clinical outcomes in HFpEF, although the relative myocardial energetic efficiency and clinical outcomes in HFpEF, although the relative contribution of individual pathways remains under investigation.

Accumulating preclinical, translational, and clinical evidence has led to the proposal that SGLT2 inhibitors may help restore aspects of mitochondrial–inflammatory homeostasis in HFpEF (Table 2). However, this concept should currently be regarded as an emerging mechanistic framework rather than a fully established causal mechanism. Experimental studies in cardiometabolic disease models suggest that SGLT2 inhibition may improve mitochondrial energy metabolism, enhance mitochondrial quality control, and reduce mitochondrial reactive oxygen species (ROS) production (68). These alterations have been associated with attenuation of inflammatory signaling, reduced activation of inflammasome-related pathways, and decreased production of downstream inflammatory cytokines (Figure 2). Collectively, these findings support the concept that metabolic and inflammatory pathways are closely interconnected, although the precise causal relationships remain incompletely understood (69).

**Table 2:** Mechanistic effects of SGLT2 inhibitors on the mitochondrial-inflammatory pathways in HFpEF.

Mechanistic domain	HFpEF pathology	Effect of SGLT2 inhibitors	Evidence type
Substrate utilization	Metabolic inflexibility; impaired FAO/ketone use	↑ ketone body utilization; improved FAO flexibility; shift toward energetically favourable fuels.	Preclinical + Clinical
Mitochondrial quality control	Impaired mitophagy and biogenesis; defective dynamics	↑ Mitophagy; ↑ mitochondrial biogenesis; enhanced fission-fusion balance	Preclinical
Oxidative stress	↑ Mitochondrial ROS; redox imbalance	↓ mtROS; improved redox balance; restoration of thiol redox homeostasis	Preclinical + Translational
Inflammatory signaling	NLRP3 inflammasome activation; chronic cytokine release	↓ NLRP3 activation; ↓ IL-1β/IL-6; attenuation of NF-kB signaling	Preclinical + Clinical correlations
Endothelial function	Coronary microvascular dysfunction; ↓NO bioavailability	↑ NO bioavailability; improved endothelial-dependent vasodilation	Clinical + Translation
Cardiac energetics	↓ATP reserve; energetic mismatch in exercise	↑ myocardial phosphocreatine/ATP ratio; improved energetics on stress tests	Clinical imaging/metabolism
Fibrosis and remodeling	Pro-fibrotic gene expression; matrix stiffening	↓ pro-fibrotic signaling (TGF-β/Smad); ↓ extracellular matrix deposition	Preclinical + post-hoc clinical
Hemodynamic load	Elevated filling pressures; ventricular stiffness	↓ preload/afterload; reduced left atrial pressure	Clinical (trial endpoints)

Clinical observations provide indirect support for this mechanistic framework, although direct mechanistic validation in humans remains limited. Several clinical studies have reported reductions in circulating inflammatory biomarkers, including interleukin-6 and high-sensitivity C-reactive protein, following treatment with SGLT2 inhibitors, although these findings have not been entirely consistent across studies. Additionally, improvements in metabolic parameters that appear to be partially independent of glucose lowering have been reported, supporting the possibility that metabolic and mitochondrial modulation contributes to the therapeutic effects of SGLT2 inhibitors beyond glycemic control, although the relative importance of these mechanisms remains uncertain (70). Improvements in myocardial energetics and vascular function have also been reported in imaging and biomarker studies. These findings are consistent with, but do not conclusively demonstrate, improved mitochondrial-inflammatory regulation (71).

This framework may partly contribute to explaining the favorable clinical outcomes observed with SGLT2 inhibitors in HFpEF, although additional mechanisms are likely to be involved. Compared with HFpEF, advanced heart failure with reduced ejection fraction (HFrEF) is generally characterized by more extensive cardiomyocyte loss and structural remodeling, which may reduce the capacity for metabolic interventions to restore functional reserve. Nevertheless, mitochondrial dysfunction, inflammatory activation, and metabolic remodeling contribute to both syndromes. In many HFpEF phenotypes, myocardial architecture remains relatively preserved, potentially allowing improvements in mitochondrial function and inflammatory regulation to translate into measurable functional benefits (72). Accordingly, restoration of mitochondrial–inflammatory coupling has been proposed as one potential mechanistic explanation for the clinical benefits of SGLT2 inhibitors in HFpEF. However, prospective mechanistic clinical studies are required to directly evaluate this hypothesis. This conceptual framework may also help guide the development of future therapeutic strategies targeting upstream immunometabolic dysfunction rather than focusing solely on downstream structural remodeling (73).

Despite the growing body of supportive evidence, several important uncertainties remain. Most mechanistic data linking SGLT2 inhibitors to mitochondrial quality control and inflammatory regulation originate from experimental models, whereas human studies have largely demonstrated associations rather than direct causality. Furthermore, the relative contribution of myocardial vs. systemic metabolic effects, the influence of HFpEF phenotypic heterogeneity, and the temporal sequence of mitochondrial and inflammatory adaptations remain incompletely understood. Addressing these questions through mechanistic clinical studies incorporating advanced metabolic imaging, tissue phenotyping, biomarker-guided patient stratification, and longitudinal functional assessment will be important for determining whether restoration of mitochondrial–inflammatory coupling contributes directly to the clinical benefits of SGLT2 inhibitors in HFpEF.

## 8 Why HFpEF responds differently than HFrEF to SGLT2 inhibition

Although HFpEF and heart failure with reduced ejection fraction (HFrEF) share several clinical manifestations, experimental and clinical evidence suggests that they differ in their predominant pathophysiological mechanisms while also exhibiting substantial biological overlap. SGLT2 inhibitors have consistently reduced the risk of heart failure hospitalization in patients with HFpEF, including those without diabetes, and have also demonstrated significant clinical benefits in HFrEF across multiple randomized clinical trials (73). However, the mechanisms underlying these benefits may differ between the two syndromes. In HFpEF, greater contributions from metabolic, mitochondrial, and immunometabolic modulation have been proposed, whereas neurohormonal and hemodynamic mechanisms remain important contributors in both HFpEF and HFrEF.

Advanced HFrEF is generally characterized by more extensive structural remodeling, including cardiomyocyte loss, replacement fibrosis, ventricular dilation, and disruption of myocardial architecture. In this setting, mitochondrial dysfunction is thought to arise predominantly from ischemic injury, sustained neurohormonal activation, oxidative stress, and impaired bioenergetic adaptation (74, 75). These structural alterations may reduce the capacity of metabolic interventions to restore myocardial energetic reserve, particularly in advanced stages of disease. Consequently, although SGLT2 inhibitors provide important clinical benefits in HFrEF, the extent to which mitochondrial modulation contributes to these effects remains uncertain and may differ from that observed in HFpEF (76).

In many cardiometabolic HFpEF phenotypes, myocardial structure remains relatively preserved despite impaired cellular energetics and reduced metabolic flexibility. Mitochondrial dysfunction in HFpEF appears to be more closely associated with metabolic stress, altered substrate utilization, redox imbalance, and chronic low-grade inflammatory signaling than with extensive irreversible myocardial injury. Experimental studies and limited analyses of human myocardial tissue have demonstrated alterations in substrate metabolism, increased mitochondrial reactive oxygen species (ROS) production, and impaired mitochondrial quality-control processes despite preserved left ventricular ejection fraction (77, 78). These characteristics may provide a greater opportunity for therapies targeting mitochondrial function and metabolic regulation to improve myocardial performance, although this hypothesis requires further mechanistic and clinical validation.

Patterns of inflammatory activation also differ between HFpEF and HFrEF, although inflammatory signaling contributes to the pathophysiology of both syndromes. In cardiometabolic HFpEF, inflammatory activation is commonly associated with obesity, insulin resistance, aging, and chronic systemic metabolic stress and is generally characterized by persistent low-grade inflammation. In contrast, inflammatory activation in HFrEF is more frequently associated with myocardial injury, neurohormonal activation, and adverse ventricular remodeling, although substantial overlap exists between the two syndromes (79). Experimental evidence further suggests that SGLT2 inhibitors may preferentially attenuate metabolically driven inflammatory pathways through improvements in mitochondrial function, redox homeostasis, and immunometabolic regulation. These observations may partly contribute to explaining the favorable clinical outcomes observed with SGLT2 inhibitors in HFpEF, including in patients without diabetes, although additional mechanisms are likely to be involved (80).

Importantly, the distinction between HFpEF and HFrEF should be regarded as relative rather than absolute. Mitochondrial dysfunction, oxidative stress, inflammatory activation, and metabolic remodeling have all been documented in both syndromes, although their relative contribution, temporal evolution, and therapeutic implications may differ. Furthermore, the marked biological heterogeneity of HFpEF complicates its current diagnostic construct, as patients meeting existing diagnostic criteria may exhibit substantially different molecular mechanisms despite similar clinical presentations. Recognizing this heterogeneity is important for interpreting therapeutic responses and supports ongoing efforts toward mechanism-based phenotyping and precision medicine. Accordingly, the proposed mitochondrial–inflammatory framework is likely to be most applicable to cardiometabolic HFpEF phenotypes rather than universally across all patients with HFpEF. Future comparative mechanistic studies integrating molecular profiling, advanced imaging, and phenotype-specific clinical characterization will be important to better define the shared and distinct biological pathways underlying therapeutic responses in HFpEF and HFrEF.

## 9 Biomarkers and patient stratification for precision therapy

**Figure 3:**
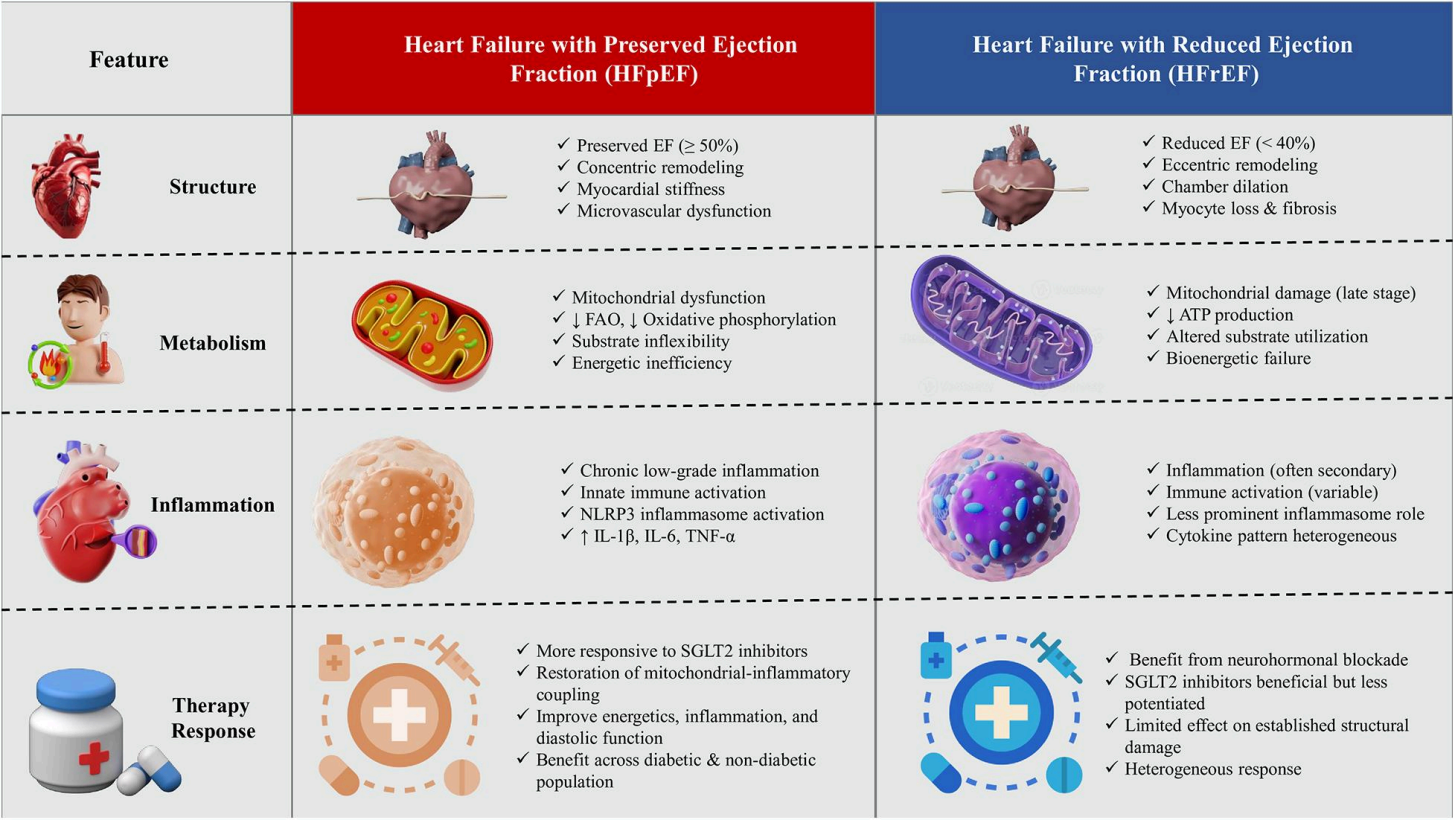
Comparative immunometabolic and structural features of HFpEF and HFrEF and their potential therapeutic implications. The schematic comparison of the predominant biological characteristics of heart failure with preserved ejection fraction (HFpEF) and heart failure with reduced ejection fraction (HFrEF). HFpEF is characterized by relatively preserved myocardial structure with prominent metabolic dysfunction, mitochondrial impairment, chronic low-grade inflammation, and microvascular dysfunction, whereas HFrEF is generally associated with more extensive cardiomyocyte loss, adverse ventricular remodeling, and replacement fibrosis. Mitochondrial dysfunction, inflammatory activation, and oxidative stress contribute to both syndromes, although their relative contributions may differ. The figure illustrates the proposed concept that the therapies targeting immunometabolic and mitochondrial pathways, including SGLT2 inhibitors, may be particularly relevant in cardiometabolic HFpEF phenotypes while acknowledging that multiple mechanisms likely contribute to therapeutic responses in both HFpEF and HFrEF (88, 89).

The proposed mitochondrial–inflammatory framework highlights several limitations of conventional heart failure biomarkers in fully capturing the biological heterogeneity of HFpEF. Conventional biomarkers primarily reflect myocardial stress and hemodynamic burden but provide limited insight into the underlying immunometabolic alterations that may influence disease progression and therapeutic response (Figure 3). Although natriuretic peptides remain clinically valuable for diagnosis, risk stratification, and prognosis, they may not adequately capture the biological heterogeneity relevant to metabolism-targeted therapeutic strategies (Table 3) (81).

**Table 3:** Biomarkers and tools for identifying proposed mitochondrial-inflammatory HFpEF phenotypes.

Biological domain	Biomarker/tool	Proposed mechanistic relevance	Level of evidence/clinical readiness
Systemic inflammation	hsCRP, IL-6, TNF-α	Chronic low-grade inflammation associated with cardiometabolic HFpEF	Established clinical biomarkers: prognostic but limited specificity for patient stratification
Inflammasome activity	IL-1β, IL-18 (circulating or stimulated assays)	Proposed indicators of NLRP3 inflammasome activation associated with mitochondrial stress	Emerging translational biomarkers: clinical validation remains limited
Metabolic substrate utilization	Ketone bodies (β-hydroxybutyrate), lactate	Reflect Myocardial substrate utilization and energetic adaptation	Clinically measurable; emerging evidence for HFpEF phenotyping
Mitochondrial metabolism	Acylcarnitine profiles	Reflect Incomplete Fatty Acid Oxidation and mitochondrial metabolic remodeling	Translational biomarkers; metabolomics platform required; prospective validation needed
Redox state/oxidative stress	NAD+/NADH-related metabolites, oxidized thiols	Proposed indicators of Mitochondrial redox imbalance	Experimental; promising mechanistic biomarkers requiring clinical validation
Fibrosis and matrix remodeling	Galectin-3, soluble ST2	Reflect Inflammation-associated extracellular matrix remodeling	Clinically available; indirect indicators with limited specificity for mitochondrial dysfunction
Cardiac structure (diffuse fibrosis)	CMR T1 mapping, extracellular volume fraction	Reflect diffuse myocardial remodeling associated with chronic metabolic and inflammatory stress	Clinically available; increasingly standardized imaging biomarkers
Myocardial energetics	^31^p-MRS (PCr/ATP ratio)	Assesses myocardial energetic efficiency	Research tools: reference standard for non-invasive myocardial energetics
Myocardial metabolism	PET (glucose, FA, oxygen utilization)	Assesses myocardial substrate utilization and metabolic flexibility	Research tool: high mechanistic resolution but limited clinical availability
Exercise-integrated metabolism	CPET with metabolic profiling	Assesses systemic energetic limitation during exercise	Clinical-research interface; potential utility for integrated phenotyping requires further validation

Several circulating inflammatory biomarkers, including high-sensitivity C-reactive protein (hsCRP), interleukin-6 (IL-6), and mediators related to tumor necrosis factor (TNF) signaling, are frequently elevated in patients with HFpEF and have been associated with disease severity and adverse clinical outcomes (2, 82). However, their limited specificity restricts their utility as stand-alone tools for patient stratification. Biomarkers reflecting metabolic remodeling and mitochondrial substrate utilization may provide complementary information regarding the underlying biological processes. These include circulating lactate, acylcarnitine profiles, and ketone body metabolites. Altered acylcarnitine profiles have been associated with impaired fatty acid oxidation and mitochondrial metabolic dysfunction in patients with HFpEF (83). In addition, biomarkers reflecting redox homeostasis and nicotinamide adenine dinucleotide (NAD⁺) metabolism have emerged as promising investigational candidates. Although these pathways are biologically consistent with proposed mechanisms of SGLT2 inhibitor action, their value for patient stratification and therapeutic guidance requires prospective clinical validation (84).

Advanced multimodality imaging provides complementary approaches for characterizing myocardial remodeling and metabolic alterations in HFpEF. Cardiac magnetic resonance (CMR) imaging with native T1 mapping and extracellular volume (ECV) quantification can identify diffuse myocardial fibrosis associated with chronic inflammation and metabolic stress (85, 86). Positron emission tomography (PET)-based metabolic imaging may enable *in vivo* assessment of myocardial substrate utilization and energy metabolism. Integrating circulating biomarkers with advanced metabolic imaging may facilitate identification of HFpEF phenotypes characterized by greater immunometabolic dysregulation and may ultimately support more individualized therapeutic strategies, although this approach requires prospective clinical validation (87).

Despite considerable progress, several challenges remain before biomarker-guided precision medicine can be implemented in routine HFpEF management. Many candidate biomarkers, including metabolomic signatures, acylcarnitine profiles, and NAD⁺-related metabolites, have primarily been evaluated in relatively small observational studies and therefore require validation in larger, prospective, multicenter cohorts. Furthermore, metabolomic analyses remain technically demanding, relatively costly, and lack standardized analytical platforms, limiting their widespread clinical implementation. Future prospective clinical studies should determine whether integrating circulating biomarkers with advanced imaging, detailed clinical phenotyping, and molecular profiling improves patient stratification and predicts therapeutic responses to SGLT2 inhibitors beyond currently established clinical risk markers.

## 10 Future direction: advancing mitochondrial-inflammatory targeting in HFpEF

The clinical benefits of SGLT2 inhibitors in HFpEF provide an opportunity to further investigate whether modulation of mitochondrial–inflammatory interactions contributes to therapeutic responses and may inform the development of future therapeutic strategies. Several important questions remain unresolved, including the relative contributions of cardiac and extracardiac tissues to therapeutic benefit, the reversibility of mitochondrial–inflammatory alterations across different stages of HFpEF, and the temporal relationship between metabolic remodeling and inflammatory activation. Future mechanistic and clinical studies should integrate advanced metabolic imaging, inflammatory profiling, biomarkers of mitochondrial quality control, and molecular phenotyping alongside conventional clinical endpoints. Such integrated study designs may facilitate biomarker-guided patient stratification and enable prospective evaluation of whether changes in mitochondrial–inflammatory pathways are associated with therapeutic responses.

Intervening before the development of advanced structural remodeling may provide greater opportunities for modifying disease progression, although this hypothesis requires prospective clinical validation. Combination strategies targeting both mitochondrial function and maladaptive inflammatory signaling represent a promising area for future investigation. Potential approaches include combining SGLT2 inhibitors with therapies that enhance mitochondrial biogenesis, preserve nicotinamide adenine dinucleotide (NAD⁺) homeostasis, or selectively modulate inflammatory pathways. However, the safety, efficacy, and mechanistic interactions of these combination strategies remain to be established in well-designed clinical studies. Future precision medicine investigations should also account for biological heterogeneity related to age, sex, metabolic status, comorbidities, and HFpEF phenotype, as these factors may influence immunometabolic regulation and therapeutic response.

Importantly, future research should prioritize prospective mechanistic clinical studies designed to distinguish association from causation. Integrating advanced cardiac imaging, multi-omics technologies, metabolomic profiling, and tissue-based analyses within randomized clinical trials may help determine whether modulation of mitochondrial–inflammatory pathways contributes directly to the clinical benefits of SGLT2 inhibitors. Such studies will also be important for validating candidate biomarkers, refining biologically informed HFpEF phenotypes, and identifying patients most likely to benefit from metabolism-targeted therapies. Until such evidence becomes available, the mitochondrial–inflammatory framework should be regarded as a promising conceptual model that guides hypothesis-driven research rather than as an established therapeutic paradigm.

## 11 Conclusion

Heart failure with preserved ejection fraction (HFpEF) is increasingly recognized as a heterogeneous multisystem syndrome in which immunometabolic disturbances, mitochondrial dysfunction, and chronic low-grade inflammation may contribute to disease development and progression, particularly in cardiometabolic HFpEF phenotypes. This conceptual framework complements traditional hemodynamic and structural models by integrating emerging evidence linking metabolic stress, mitochondrial dysfunction, and inflammatory signaling while providing one possible explanation for the biological heterogeneity of HFpEF and its close association with metabolic comorbidities. Within this context, mitochondrial–inflammatory uncoupling has been proposed as a conceptual mechanistic framework linking systemic metabolic disturbances with myocardial dysfunction in selected HFpEF phenotypes. This perspective suggests that interventions targeting upstream immunometabolic processes may have therapeutic potential in biologically defined patient subgroups, although this hypothesis requires further mechanistic and clinical validation.

Within this evolving framework, sodium–glucose cotransporter 2 (SGLT2) inhibitors represent the first pharmacological class to demonstrate consistent clinical benefits across a broad spectrum of patients with HFpEF. Accumulating experimental and translational evidence suggests that, beyond glucose lowering, SGLT2 inhibitors may influence mitochondrial energy metabolism, enhance mitochondrial quality control, and attenuate metabolically driven inflammatory signaling across multiple cardiovascular cell types. However, direct mechanistic confirmation of these effects in patients with HFpEF remains limited, and the relative contribution of mitochondrial–inflammatory modulation to the overall clinical benefits of SGLT2 inhibitors has yet to be established. These findings therefore provide a biologically plausible, rather than definitive, explanation for the favorable clinical outcomes observed in both diabetic and non-diabetic patients with HFpEF, while acknowledging that multiple complementary mechanisms are likely to contribute.

Looking forward, integrating mitochondrial biology, immunometabolism, biomarker discovery, advanced metabolic imaging, and multi-omics approaches may facilitate more precise biological phenotyping of HFpEF and support the development of mechanism-informed therapeutic strategies. Future prospective mechanistic clinical studies will be important to determine whether modulation of mitochondrial–inflammatory pathways contributes directly to therapeutic responses, to validate candidate biomarkers, and to identify patient subgroups most likely to benefit from metabolism-targeted interventions. Until such evidence becomes available, mitochondrial–inflammatory uncoupling should be regarded as an evolving conceptual framework that informs hypothesis-driven research rather than an established pathogenic mechanism or therapeutic paradigm.
